# Resting-state EEG spectral characteristics before and after a single auditory integration training session in children with autism spectrum disorder

**DOI:** 10.3389/fpsyt.2026.1948611

**Published:** 2026-10-28

**Authors:** Yao Wang, Yiying You, Xiaowen Wu, Haozhe Chu, Chong Lu

**Affiliations:** 1 Key Laboratory of Language and Cognitive Science (Ministry of Education), Beijing Language and Culture University, Beijing, China; 2 Institute of Life and Health Sciences, Beijing Language and Culture University, Beijing, China; 3 KeyLaboratory of Language and Cognitive Science (Ministry of Education), Beijing Language and Culture University, Beijing, China; 4 College of International Education, Minzu University of China, Beijing, China

**Keywords:** aperiodic exponent, auditory integration training, autism spectrum disorder, neural oscillations, resting-state EEG, spectral parameterization

## Abstract

**Background:**

Resting-state EEG characteristics may differ between autistic and non-autistic children. Conventional band power mixes periodic and broadband activity; spectral parameterization separates periodic peaks from the aperiodic background. Auditory integration training (AIT) is used as a complementary intervention, but its immediate neurophysiological and clinical effects remain uncertain. We examined baseline EEG differences and pre-post changes around one AIT session.

**Methods:**

Twenty children with ASD and 15 typically developing (TD) children aged 6–8 years participated. Resting EEG was recorded in ASD before and immediately after a 30-min Berard AIT session. Sixteen ASD participants had usable paired recordings. Relative power was measured in five bands. Periodic and aperiodic components were parameterized with FOOOF. Sensitivity analyses varied R-squared threshold, aperiodic mode, fitting settings, channel aggregation.

**Results:**

At baseline, ASD showed higher delta and lower theta, alpha, and beta relative power than TD. After modeling peaks, aperiodic exponent and offset did not differ between groups. Among 16 paired ASD recordings, delta decreased and beta/gamma increased post-session; paired tests of exponent and offset were nonsignificant. Five of 20 baseline and seven of 18 post-session recordings had R-squared<0.90, but sensitivity analyses did not change null aperiodic findings.

**Conclusions:**

This uncontrolled pilot study found immediate pre-post band-power differences around one Berard AIT session but no significant FOOOF-derived aperiodic changes. Findings are hypothesis-generating and cannot distinguish AIT-specific effects from repeated measurement, arousal, expectancy, or generic auditory stimulation. Variable fit quality and small sample limit precision; controlled longitudinal studies are needed.

## 1 Introduction

Autism spectrum disorder (ASD) is defined by differences in social communication and restricted or repetitive patterns of behavior. Resting-state EEG provides a non-invasive description of spontaneous neural activity and allows comparison of spectral characteristics across participants and recording conditions. Studies have reported group-level differences in resting-state oscillatory activity between autistic and non-autistic participants (1–3), but the functional meaning of these EEG differences is not established and is likely to vary across individuals and developmental stages. One proposed mechanistic account is the excitation-inhibition (E/I) imbalance hypothesis (4, 5). This hypothesis suggests that variation in excitatory and inhibitory processes may influence neural variability and information processing; however, group-level scalp EEG differences do not directly demonstrate altered E/I balance, functional impairment, or a mechanism specific to ASD. It also remains unclear whether the observed EEG characteristics are stable across time or can vary after external stimulation. A measurable EEG change after stimulation would indicate short-term neurophysiological variation, but it would not by itself indicate normalization or clinical benefit. Accordingly, combining auditory stimulation with EEG may help describe short-term spectral variation, provided that mechanistic and clinical interpretations remain separate from the observed signals.

Conventional band-power analysis cannot fully distinguish rhythmic oscillations from the underlying broadband spectral background. Spectral parameterization separates periodic and aperiodic components and may therefore provide complementary descriptions of EEG spectra (6). The aperiodic component, commonly characterized by a 1/f-like profile, varies with developmental stage, cognitive state, vigilance, and neurological condition (6–8). Its exponent describes the frequency-dependent decay of power, with lower values indicating a flatter slope and relatively greater high-frequency contributions. Aperiodic parameters have been investigated as candidate correlates of E/I-related processes and cortical gain (9), but scalp-derived exponent and offset are not direct measurements of these mechanisms. Developmental studies have associated aperiodic EEG activity with later autism-related outcomes and language development (10–12), and periodic and aperiodic characteristics have been compared across physiological states such as sleep (13). These observations support including aperiodic analysis alongside conventional band power, while treating the resulting parameters as descriptive spectral measures rather than established measures of functional impairment or treatment response. If auditory exposure changes aperiodic parameters, that finding would require controlled replication and independent functional validation before it could support a mechanistic or clinical interpretation. Whether such short-term changes occur in autistic children remains unknown.

Despite extensive investigation of EEG differences in ASD, several questions remain unresolved. Studies have reported differences in resting-state delta, alpha, and beta activity, but findings vary with developmental stage, clinical characteristics, recording conditions, and analytical approach (1–3). Recent pediatric studies have also described group differences in alpha modulation and microstate dynamics (2, 14). The developmental characteristics of aperiodic activity during the early school years remain insufficiently understood. Auditory integration training (AIT) has been used as a complementary intervention for autistic children since the 1990s. Its rationale is that modified auditory stimulation may influence sensory processing through repeated engagement of auditory systems. Evidence for clinical efficacy remains mixed, and systematic reviews identify substantial between-study variability and limited high-quality evidence of behavioral benefit (15, 16). Preliminary studies have also examined AIT combined with repetitive transcranial magnetic stimulation (17), but those findings do not establish the effect of AIT alone. Earlier electrophysiological studies of auditory-based interventions in ASD focused mainly on evoked responses or behavioral outcomes (18, 19). Research in other samples has reported auditory training-related changes in subcortical encoding, temporal precision, and other neurophysiological measures (20–23); such changes cannot be assumed to represent benefit or to generalize to resting-state activity in autistic children. To our knowledge, no study has tested immediate resting-state aperiodic EEG differences around one AIT session in this population. The present question was therefore descriptive: whether relative band power or aperiodic parameters differed between recordings obtained immediately before and after the session. Because the study lacked a control session and behavioral or functional outcome measures, any EEG difference could not establish AIT specificity, intervention-related neural plasticity, or clinical benefit. Recent computational work positions EEG within multimodal ASD/ADHD classification pipelines (24, 25), and studies in other neurological conditions illustrate developments in explainable learning, feature fusion, and cross-session adaptation (26–29). These reports provide methodological context but do not constitute evidence of AIT efficacy or biomarker validity in ASD.

The present pilot study examined baseline EEG differences between ASD and TD children and immediate pre-post EEG differences in ASD children around one AIT session. We evaluated relative band power and peak-separated aperiodic parameters. We did not test whether individual changes in aperiodic and oscillatory measures were associated, and we did not include a sham-AIT or TD-AIT arm. Accordingly, the study cannot test coordinated modulation, AIT specificity, or ASD specificity. Children aged 6–8 years were studied as a defined early school-age sample; the developmental range should not be considered homogeneous. All intervention-related inferences are exploratory.

## 2 Method

### 2.1 Subjects

A total of 35 children participated, including 20 children with autism spectrum disorder (ASD) and 15 typically developing (TD) children. All participants were aged 6–8 years, were native Mandarin speakers, and were right-handed. The ASD group comprised 20 children (mean age = 7.4 years; 15 male) and the TD group comprised 15 children (mean age = 7.1 years; 10 male). Children with ASD had been clinically diagnosed by certified clinicians at a tertiary Grade A hospital according to DSM-5 criteria. Participants were recruited through public advertisements at a local research institution. Written informed consent was obtained from parents or legal guardians. The available study record does not document a separate written child-assent procedure. Participants received monetary compensation. Standardized IQ, language, symptom-severity, medication, sleep, time-of-day, and individual post-attrition demographic data were not available. Eighteen post-session recordings were available; two were excluded during revision quality control, leaving 16 usable pre-post pairs. No *a priori* power calculation was performed, and the study was interpreted as exploratory.

### 2.2 Experimental design and AIT

All children with ASD received one 30-min auditory-stimulation session based on the Berard AIT method using the Earducator system (Hollagen Designs CC, Western Cape, South Africa). The same trained experimenter administered the session in a quiet laboratory. Participants listened passively to Earducator-processed Mozart music through closed headphones at approximately 70 dB SPL. Resting-state EEG was acquired immediately before and after the session using the same acquisition conditions; no additional stimulation, rehabilitation, or behavioral intervention was delivered between recordings. The study used neither a sham condition nor a TD-AIT condition, and TD children did not receive AIT. Consequently, the paired ASD comparison cannot separate effects of AIT from repeated recording, time, arousal, expectancy, or generic auditory stimulation, and it cannot determine whether any observed pattern is ASD-specific. The experimenter’s blinding status with respect to the hypotheses was not documented in the available study record.

### 2.3 EEG acquisition and analysis

EEG data were recorded using a 65-channel HydroCel Geodesic Sensor Net (Electrical Geodesics Inc., Eugene, OR, USA) with saline-based electrodes. Electrode impedances were maintained below 50 kΩ throughout data collection. Continuous EEG signals were sampled at 1,000 Hz, online band-pass filtered between 0.1 and 100 Hz, and referenced to the vertex electrode (Cz) during acquisition. Resting-state EEG was recorded for 5 min before and immediately after the AIT session. Participants were instructed to remain awake, relaxed, and minimize head and body movements during EEG acquisition. The same recording procedures and environmental conditions were maintained for both pre- and post-intervention EEG assessments.

Offline EEG preprocessing was performed using EEGLAB in MATLAB. Continuous EEG signals were band-pass filtered from 0.5 to 45 Hz, visually inspected, cleaned with ICA to remove ocular and other non-neural components, and average-referenced before spectral analysis. All saved final datasets contained the same 65 labeled electrodes. Reinspection of the final EEGLAB files recovered retained recording durations of about 5 min. The ICA decompositions retained in the final datasets contained 57–60 components. Of 18 post-session files, ID 15 was excluded because of an extreme recording duration and a robust-MAD offset outlier, and ID 18 because of a robust-MAD offset outlier; 16 paired recordings remained for primary inference.

Frequency-domain analyses were performed on the artifact-corrected continuous data using Welch power spectral density (PSD) estimation. The temporal mean was removed from each channel segment. All EEG data were resampled to 500 Hz before PSD estimation. PSD was calculated separately for every electrode using a periodic 2-s Hann window, 50% overlap, and a 1,000-point FFT, yielding a 0.5-Hz frequency resolution. Both implementations therefore yielded a 0.5-Hz frequency grid. No additional notch filter was applied during spectral recomputation; the stored data had already been filtered to 0.5–45 Hz, and analyses were restricted to 1–45 Hz. One-sided power-density estimates were used. Relative power was calculated for delta (1–4 Hz), theta (4–8 Hz), alpha (8–13 Hz), beta (13–30 Hz), and gamma (30–45 Hz). For each electrode, band power was integrated across the corresponding frequency range and divided by total power from 1 to 45 Hz.


Relative powerband=powerbandpower1−45Hz

Global relative power was calculated by averaging relative power values across all 65 electrodes for each frequency band.

Periodic and aperiodic components were parameterized using the Fitting Oscillations and One-Over-F (FOOOF) algorithm described and validated by Donoghue et al. (7), implemented with FOOOF version 1.1.1 through the official MATLAB interface. For each recording, electrode-level Welch spectra were averaged pointwise across the 65 electrodes in linear power units to form a whole-scalp spectrum P(f); averaging before the logarithm preserves the arithmetic mean power contribution of the electrodes. The positive linear spectrum was passed to FOOOF, which internally represents log10 power as the sum of an aperiodic function and N Gaussian peak functions, as specified below. In fixed mode, b is the aperiodic offset and chi is the positive aperiodic exponent; each peak has height a_n above the aperiodic fit, center frequency c_n, and bandwidth parameter w_n. The algorithm estimates an initial aperiodic background, subtracts it to flatten the spectrum, iteratively detects residual maxima meeting the height and threshold criteria, fits and removes Gaussian peaks, refits the aperiodic component, and jointly optimizes the combined model by least squares (7). Primary fits covered 1–45 Hz, used peak-width limits of 1–12 Hz, at most six peaks, a minimum peak height of 0.10 log10 power, a peak threshold of 2.0 standard deviations, and fixed aperiodic mode. The 1-Hz lower peak width was twice the 0.5-Hz frequency resolution, as recommended to avoid fitting single-bin fluctuations; the maximum peak count, minimum height, and threshold were based on validated FOOOF settings (7). These settings were selected before the revised analyses but were not preregistered. No recording reached the six-peak cap. Fixed mode was retained because knee-mode sensitivity fits produced no finite positive knee frequency in any recording and did not consistently improve fit error. Individual R-squared, mean absolute error (MAE), root-mean-square error (RMSE) in log10 power, and peak count were exported. R-squared below 0.90 triggered visual review of the observed spectrum, full model, and residuals rather than automatic exclusion. All 53 fits were reviewed; low values reflected residual curvature or high-frequency mismatch without numerical failure. Otherwise eligible low-R-squared recordings were retained in the primary analysis to avoid differential *post hoc* exclusion, and R-squared thresholds of 0.80 and 0.90 were examined in sensitivity analyses. Additional sensitivity analyses used knee mode; a 2-40-Hz fit range; peak widths of 1–8 Hz; a minimum peak height of 0.05; a 1.5-standard-deviation peak threshold; eight maximum peaks; the across-channel median in linear power; the geometric mean.


$$\log_{10}P\left(f\right)=b-\text{χ}\log_{10}f+\overset{N}{\underset{n=1}{\sum }}a_{n}\exp\left[-\frac{{\left(f-c_{n}\right)}^{2}}{2w_{n}^{2}}\right]$$

### 2.4 Statistical analysis

All statistical analyses were performed using Python implementations for the revision analyses. Baseline comparisons used ASD baseline (n = 20) and TD (n = 15) data. Relative power was analyzed with a 2 x 5 mixed ANOVA (Group x Frequency Band), followed by independent-samples t-tests. The paired ASD analysis used 16 quality-controlled pairs and a Time x Frequency Band repeated-measures ANOVA followed by paired t-tests. Benjamini-Hochberg FDR correction was applied separately to the five band-level *post hoc* comparisons following each ANOVA (five baseline group comparisons and five paired pre-post comparisons); omnibus ANOVA p values were not included in these correction families. Aperiodic exponent and offset were prespecified parameters and were tested directly without FDR correction. Normality was assessed using the Shapiro-Wilk test. Baseline variables satisfying normality in both groups were analyzed with independent-samples t-tests; otherwise, the Mann-Whitney U test was used. For pre-post comparisons, normally distributed difference scores were analyzed with paired t-tests; otherwise, the Wilcoxon signed-rank test was used. Mean differences, confidence intervals, paired t statistics, and Cohen’s dz were defined consistently as post minus pre; baseline contrasts were defined as ASD minus TD. Fit-quality metrics were compared with Mann-Whitney U tests at baseline and Wilcoxon signed-rank tests for paired recordings; within each contrast, the R-squared, MAE, and RMSE tests formed one three-test FDR family. Attrition comparisons and parameter/specification sensitivity analyses were exploratory and unadjusted. Sphericity was assessed with Mauchly’s test, and Greenhouse-Geisser correction was applied where the assumption was violated. We report partial eta squared for ANOVA models, Cohen’s d or dz for t-tests, and 95% confidence intervals for raw mean differences. No correlation, mediation, or shared-variance analysis was performed between individual aperiodic and band-power changes. FDR-adjusted p< 0.05 was considered significant for the band-level and fit-quality families; two-sided p< 0.05 was used for direct aperiodic tests.

## 3 Results

### 3.1 Baseline spectral differences between groups

Baseline comparisons showed different relative-power distributions between the ASD and TD groups. For global relative power, the mixed-design ANOVA showed a significant Group × Frequency Band interaction, F(4,132) = 22.449, p< 0.001, ηp² = 0.405, indicating that the distribution of relative power across frequency bands differed between groups. The main effect of Group was not significant, F(1,33) = 1.335, p = 0.256, whereas the main effect of Frequency Band was significant, F(4,132) = 782.105, p< 0.001, ηp² = 0.972.

FDR-corrected *post hoc* comparisons showed significant group differences in relative power across several frequency bands. Compared with TD participants, the ASD group showed higher delta relative power (ASD: 0.763 +/- 0.022; TD: 0.606 +/- 0.022; t(33) = 4.975, FDR-p< 0.001, Cohen’s d = 1.70) and lower theta (ASD: 0.096 +/- 0.009; TD: 0.164 +/- 0.013; t(33) = -4.480, FDR-p< 0.001, d = -1.53), alpha (ASD: 0.051 +/- 0.007; TD: 0.118 +/- 0.013; t(33) = -5.059, FDR-p< 0.001, d = -1.73), and beta relative power (ASD: 0.054 +/- 0.006; TD: 0.078 +/- 0.006; t(33) = -2.700, FDR-p = 0.014, d = -0.92). Gamma relative power did not differ significantly between groups (FDR-p = 0.725). Grand-average PSD profiles are shown in **Figure 1**. These comparisons describe group-level spectral differences and do not, by themselves, establish functional impairment or a mechanism.

**Figure 1:**
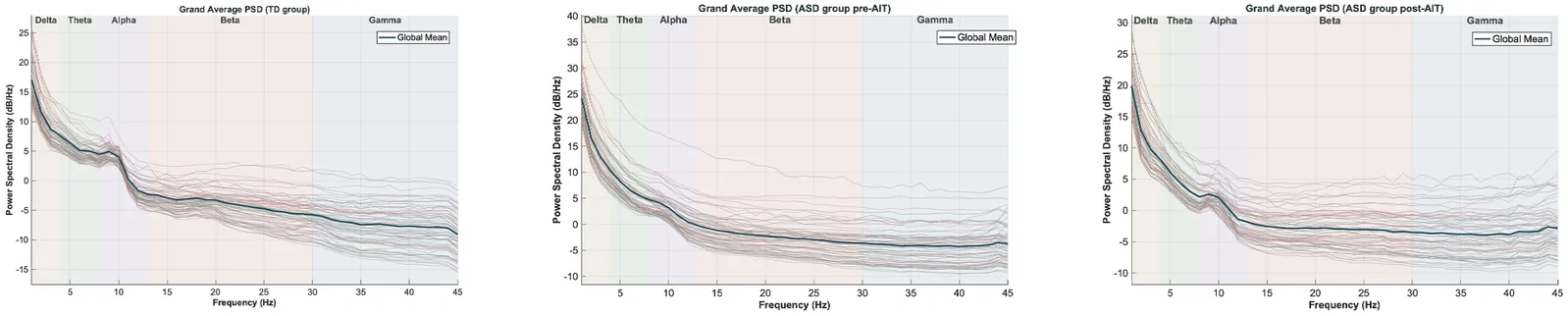
Grand-average power spectral density (PSD) across groups. Grand-average EEG PSD curves are shown for children with ASD before AIT, children with ASD after AIT, and TD children.

### 3.2 Baseline aperiodic spectral parameters

The baseline aperiodic exponent satisfied the normality assumption in both groups and did not differ significantly between ASD and TD children (ASD: 1.273 +/- 0.117; TD: 1.420 +/- 0.090; ASD-TD mean difference = -0.147, 95% CI -0.464 to 0.170; t(33) = -0.943, p = 0.353, Cohen’s d = -0.32). Baseline offset was non-normally distributed in the ASD group (Shapiro-Wilk W = 0.884, p = 0.021) and was therefore analyzed using the Mann-Whitney U test. Offset did not differ significantly between groups (ASD median = 1.933, IQR = 1.718-2.368; TD median = 1.989, IQR = 1.529-2.190; U = 162, p = 0.701). Thus, after periodic peaks were modeled, the data did not support a baseline group difference in either aperiodic parameter. Individual observations and group summaries are shown in **Figures 2A, B**.

**Figure 2:**
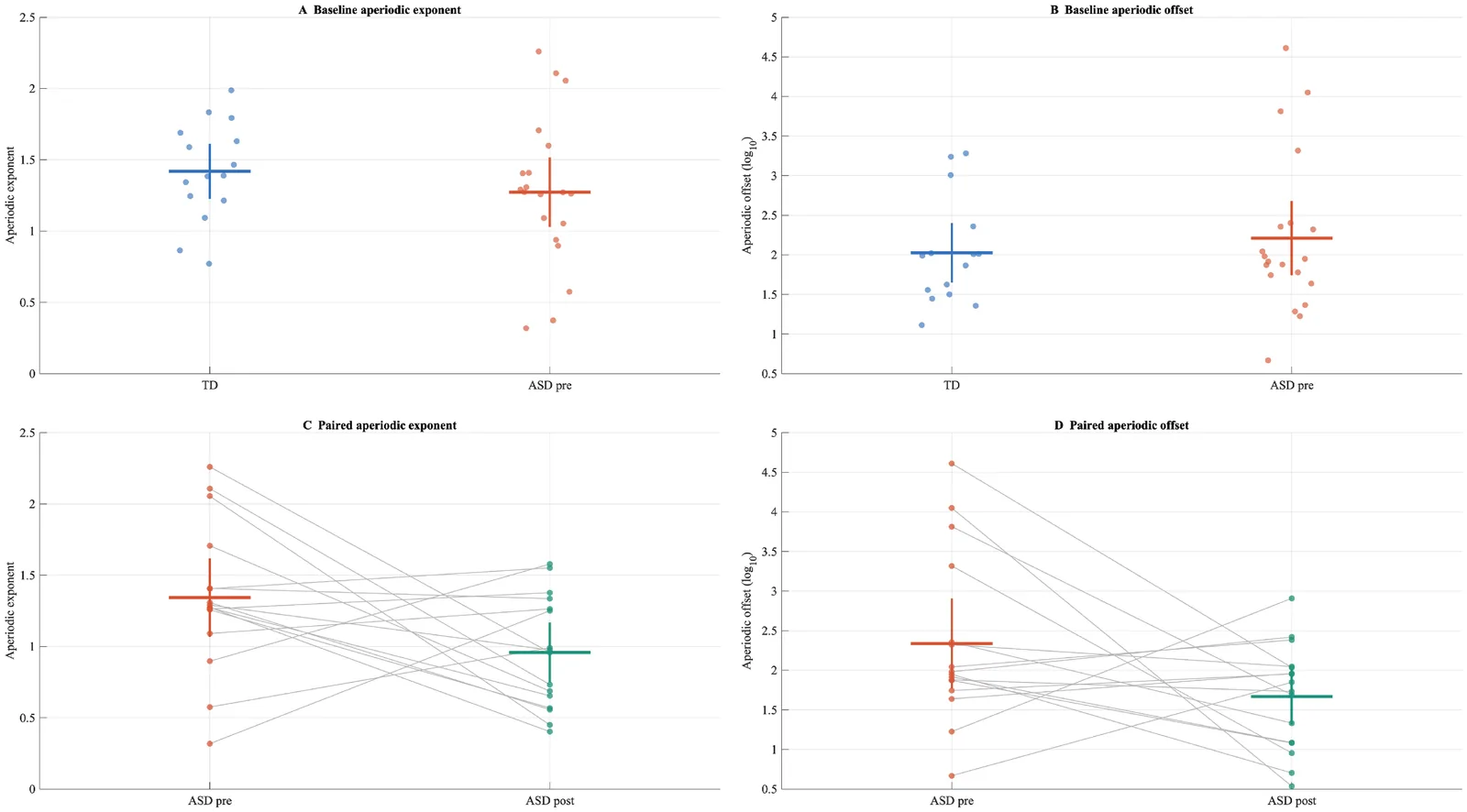
Individual and group-level distributions of FOOOF-derived aperiodic parameters. Panels **(A, B)** show individual baseline observations for TD children (n = 15) and children with ASD (n = 20). Panels **(C, D)** show the 16 quality-controlled ASD pre-post pairs; gray lines connect repeated observations from the same participant. Horizontal bars indicate means and vertical bars indicate 95% confidence intervals. Two post-session records were excluded during revision quality control.

### 3.3 Immediate paired differences in relative power

Among the 16 ASD participants with usable paired recordings, relative power showed a Time x Frequency Band interaction, F(4,60) = 5.699, p< 0.001, partial eta squared = 0.160; the main effect of Time was not significant (p = 0.745). FDR-corrected paired comparisons showed lower delta relative power after the session (mean post-pre difference = -0.079, 95% CI -0.142 to -0.016; t(15) = -2.687, FDR-p = 0.028, Cohen’s dz = -0.67) and higher beta (mean difference = 0.027, 95% CI 0.007 to 0.047; t(15) = 2.827, FDR-p = 0.028, dz = 0.71) and gamma relative power (mean difference = 0.025, 95% CI 0.010 to 0.040; t(15) = 3.510, FDR-p = 0.016, dz = 0.88). Theta and alpha did not show significant pre-post differences. All signs follow the post-minus-pre convention. These paired-session findings do not identify their cause.

### 3.4 Immediate paired differences in aperiodic parameters

Among the 16 quality-controlled ASD pairs, the FOOOF-derived aperiodic exponent was numerically lower after the session (pre: 1.343 +/- 0.129; post: 0.958 +/- 0.099; mean post-pre difference = -0.385, 95% CI -0.790 to 0.021; t(15) = -2.022, p = 0.061, Cohen’s dz = -0.51). Offset was also numerically lower (pre: 2.337 +/- 0.267; post: 1.667 +/- 0.166; mean difference = -0.670, 95% CI -1.431 to 0.091; t(15) = -1.876, p = 0.080, dz = -0.47). Neither direct paired comparison met the two-sided p< 0.05 significance criterion. These estimates do not establish an AIT effect or an absence of aperiodic change.

### 3.5 Attrition and quality-control comparison

The 16 included ASD participants and the four participants without a usable paired observation (two without a post-session record and two excluded during revision quality control) did not differ significantly at baseline in aperiodic exponent (included: 1.343 +/- 0.129; unavailable/excluded: 0.991 +/- 0.251; t(18) = 1.225, p = 0.236, d = 0.68) or offset (included: 2.337 +/- 0.267; unavailable/excluded: 1.707 +/- 0.255; t(18) = 1.130, p = 0.273, d = 0.63). These exploratory comparisons cannot exclude attrition or quality-control selection bias because baseline band power, age, sex, symptom severity, and other relevant factors were not compared.

### 3.6 Spectral fit quality and robustness analyses

Individual fit-quality distributions are shown in **Figure 3**. R-squared was below 0.90 for 0/15 TD baseline, 5/20 ASD baseline, and 7/18 ASD post-session recordings; 4/20 ASD baseline and 4/18 post-session recordings were below 0.80. Six of the 16 primary ASD pairs had R-squared at least 0.90 at both time points. No record reached the six-peak limit. At baseline, group comparisons of R-squared, MAE, and RMSE were not significant after FDR correction (all adjusted p = 0.075); paired pre-post comparisons were also nonsignificant (all adjusted p = 1.000). Restricting both observations to R-squared >= 0.80 left 10 pairs and produced p = 0.063 for exponent and p = 0.285 for offset; the R-squared >= 0.90 analysis left six pairs and produced p = 0.303 and p = 0.557, respectively. The corresponding baseline threshold analyses were also nonsignificant (all p >= 0.431). Knee-mode fits yielded no finite positive knee frequency in any recording, and all knee-mode baseline and paired tests were nonsignificant. Results also remained nonsignificant across the alternative fit range, peak settings, channel-median and geometric-mean aggregation (all p >= 0.050).

**Figure 3:**
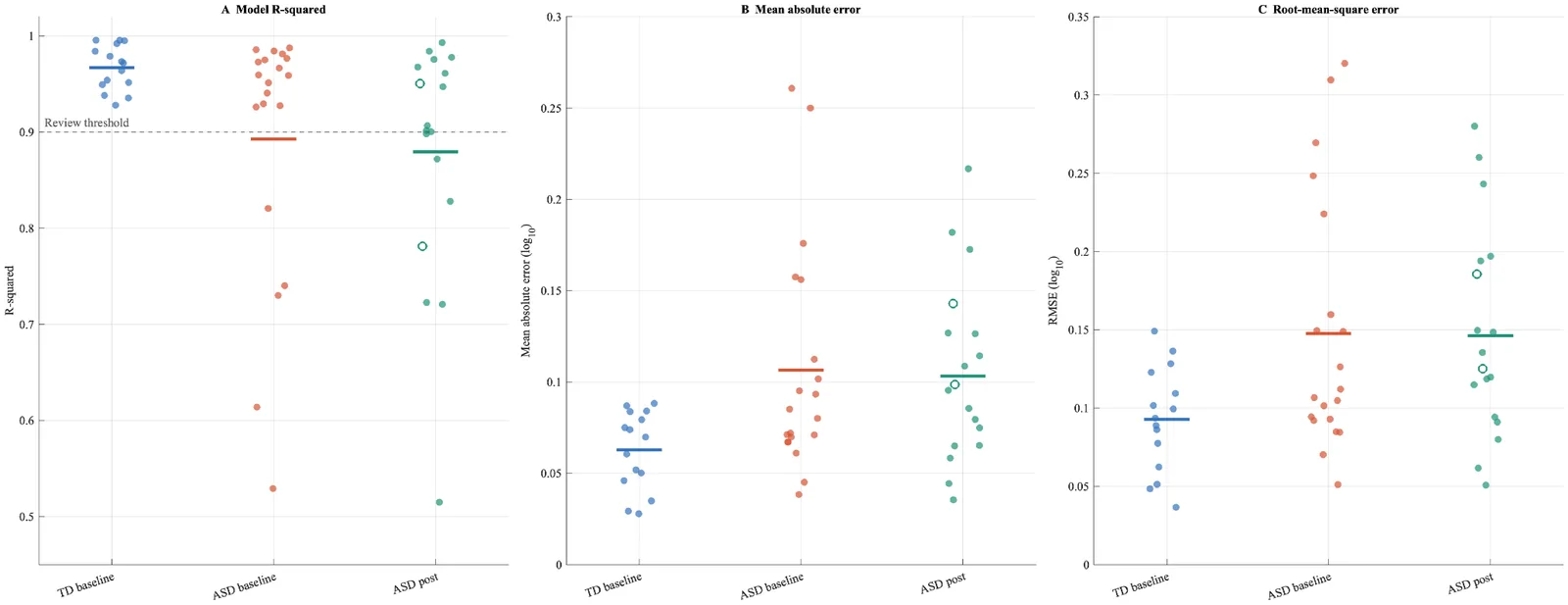
Individual FOOOF goodness-of-fit diagnostics. Panels **(A–C)** show R-squared, mean absolute error (MAE), and root-mean-square error (RMSE) for every TD baseline, ASD baseline, and ASD post-session recording. Points are individual recordings and horizontal bars are group means. The dashed line in panel **(A)** marks the prespecified R-squared = 0.90 visual-review threshold. Open post-session symbols identify IDs 15 and 18, which were excluded from the primary paired analysis for the revision-stage criteria described in Methods; low R-squared alone did not determine primary inclusion.

## 4 Discussion

This exploratory study compared baseline resting-state EEG in autistic and non-autistic children and assessed immediate paired differences around one AIT session. The baseline groups differed in the distribution of relative power across canonical bands, and selected relative-power measures differed between the two ASD recordings. Higher baseline delta relative power and lower theta, alpha, and beta relative power are broadly compatible with the heterogeneous ASD spectral-power literature (1–3), but these findings should not be interpreted as separate indicators of impaired function. Relative power is compositional: all band fractions share the same 1-45-Hz denominator, so a larger low-frequency contribution can mechanically reduce other proportions even when their absolute power does not decrease. The pattern may reflect oscillatory organization, maturation, vigilance, global spectral shape, or normalization effects, which cannot be separated by relative power alone. Moreover, the uncontrolled design does not identify AIT as the cause of any paired band-power difference, and EEG change alone does not demonstrate functional improvement or clinical benefit.

The absence of a baseline group difference in exponent or offset after spectral parameterization is not contradictory to the relative-power findings. Canonical band power integrates oscillatory peaks and the aperiodic background, and an unparameterized log-log line can be tilted or displaced by prominent peaks (7); separating peaks can therefore remove an apparent global-slope contrast. Prior developmental findings are heterogeneous: a study of preschool-aged autistic children reported higher offset but comparable slope (11), longitudinal infant work linked developmental aperiodic change to later autism and language outcomes (12), and childhood studies indicate that exponent and offset vary with age, recording condition, and scalp location (30). The present whole-scalp average may have attenuated regional effects, while the narrow age range, variable vigilance, clinical heterogeneity, and small sample further limited sensitivity. Fit quality was also less uniform in ASD: five baseline ASD spectra, but no TD spectra, had R-squared below 0.90. Nevertheless, baseline differences in R-squared, MAE, and RMSE did not survive correction, excluding low-R-squared fits at thresholds of 0.80 or 0.90 did not reveal a group effect, and alternative model settings and channel aggregation produced the same null conclusion. Thus, the result is not readily explained by one arbitrary fit choice, but it shows only that a difference was not detected; it neither establishes equivalence nor supports a direct inference about synaptic excitation-inhibition balance or neuronal firing (7–9).

The lack of a significant immediate pre-post difference in exponent or offset is also plausible after a single 30-min session. Individual PSD profiles and parameterized features can show substantial short-term stability in children with ASD and TD children (31), whereas brief auditory exposure may more readily affect transient synchronization, desynchronization, vigilance, or movement-related spectral content than the broadband background. Aperiodic estimates also carry substantial uncertainty when fit quality is limited: seven of 18 post-session spectra had R-squared below 0.90, and only six of the 16 primary pairs had R-squared at least 0.90 at both time points. Even so, paired fit-quality metrics were not detectably different, R-squared-threshold analyses remained nonsignificant, and conclusions were unchanged with knee mode, alternative fitting parameters, median or geometric channel aggregation. The relative-power findings and null aperiodic tests do not demonstrate a dissociation. Relative power is calculated from the complete PSD and therefore combines peaks, aperiodic power, and normalization by total power. Developmental data indicate that relative theta and beta power can be partly explained by aperiodic activity, whereas relative alpha power may track periodic activity more closely (32), and periodic and aperiodic measures can remain correlated after parameterization (30). The observed lower delta and higher beta and gamma proportions may therefore reflect redistributed oscillatory or normalized power, but peak-specific power, frequency, and bandwidth were not tested, and no within-participant association or formal between-effect comparison was performed. Repeated measurement, nonspecific auditory exposure, expectancy, arousal, or residual artifact remain alternative explanations.

Several limitations constrain interpretation and define the next steps. The paired analysis included 16 ASD recordings; two participants lacked post-session recordings and two records were excluded using revision-stage rather than preregistered rules. No *a priori* power calculation was performed, confidence intervals were wide, and the high-fit sensitivity sets were very small. Although extensive sensitivity analyses did not change the aperiodic conclusions, low R-squared values in several ASD recordings limit confidence in individual parameters. Whole-scalp averaging obscured regional effects, relative power introduced dependence among bands, and absolute power, peak-specific measures, behavior, and no-intervention test-retest data were unavailable. Age covered 6–8 years, sex was unbalanced, relevant cognitive and clinical covariates and blinding records were unavailable, and the study lacked sham-AIT, passive-auditory, TD-AIT, and delayed follow-up conditions. Future studies should preregister preprocessing, fit criteria, model mode, and exclusion rules; preserve complete preprocessing audit trails; justify sample size; randomize participants to active and matched control conditions; and acquire repeated and longitudinal measurements. They should jointly report absolute and relative power, aperiodic-adjusted peak power, peak frequency and bandwidth, regional parameterization, uncertainty and goodness-of-fit, within-person periodic-aperiodic associations, and formal comparisons of standardized effects, linked to sensory, language, symptom, and functional outcomes. These safeguards are required before either analysis family can be evaluated as a candidate measure of AIT response.

## 5 Conclusion

In this exploratory uncontrolled pilot, autistic children showed a different baseline relative-power distribution from TD children, and selected relative-power bands differed between the two ASD recordings. Because peak-specific periodic components were not directly examined, the analyses cannot determine which spectral features carried the band-power differences. Aperiodic exponent and offset did not differ significantly at baseline or between the paired recordings. These null findings may reflect the absence of an effect, limited statistical power, variability in fit quality, or a single session that was insufficient to produce a detectable difference. The observed EEG differences do not establish AIT-specific neural change, intervention-related neural plasticity, normalization, or clinical benefit; no behavioral or functional improvement was assessed. Controlled, adequately powered longitudinal studies with preregistered quality-control procedures and clinically relevant outcomes are needed to determine whether any frequency-specific differences are reproducible and meaningful.

## Data Availability

The raw data supporting the conclusions of this article will be made available by the authors, without undue reservation.
